# Using Re-scaled Resilience Screening Index Results and Location Quotients for Socio-Ecological Characterizations in U.S. Coastal Regions

**DOI:** 10.3389/fenvs.2019.00096

**Published:** 2019-06-19

**Authors:** Lisa M. Smith, Linda C. Harwell, J. Kevin Summers, Justin Bousquin, Kyle D. Buck, James E. Harvey, Michelle McLaughlin

**Affiliations:** 1 National Health and Environmental Effects Laboratory, Gulf Ecology Division, U.S. Environmental Protection Agency, Office of Research and Development, Gulf Breeze, FL, United States; 2 Student Services Contractor, Oak Ridge Associated Universities, Oak Ridge, TN, United States

**Keywords:** climate resilience screening index (CRSI), U.S. coastal and coastal shoreline counties, natural hazard events, location quotient (LQ), ocean economy, socio-ecological characterizations

## Abstract

In terms of natural hazard events, resilience characterizations provide a means of identifying risk profiles, degrees of preparedness, and the ability of communities to respond and recover. While nationally consistent measures of community resilience to natural hazards are needed to address widespread socio-ecological impacts from a broad policy perspective, geographically specific resilience characterizations are needed to target local resources to increase community resilience. The Climate Resilience index (CRSI) was developed to characterize the resilience of socio-ecological systems in the context of governance and risk to natural hazard events for all U.S. counties for the years 2000–2015. Those resilience characterizations were based on the full range of nationwide county domain scores. This paper presents a re-scaled application of CRSI, where county domain scores are limited to the range of scores within a specific set of U.S. coastal and shoreline counties within each of eight coastal regions. The re-scaled CRSI values for selected counties/parishes in the Gulf of Mexico (GOM) region are also presented in conjunction with calculated Location Quotients (LQ) values >1.0, which represent a high employment dependence on ocean economy sectors. Using a combination of re-scaled CRSI and LQ values provides a more holistic picture of vulnerability and resilience in these U.S. coastal shoreline counties. The relative resilience assessments presented for coastal regions are useful in identifying potential strengths and weaknesses in resilience aspects given similar hazard profiles, a signature otherwise diluted in nation-wide county-level assessments. The unique approach of combining CRSI and LQ for characterizing natural hazard resilience described could be transferred to other specific geographies as defined by population groups, hazard profiles and economic dependence.

## INTRODUCTION

Resilience describes the ability of a system and its component parts to anticipate, absorb, accommodate or recover from the effects of a hazardous event in a timely and efficient manner (Intergovernmental Panel on Climate Change (IPCC), 2012). Within the framework of resilience, sustainability is reflected in the ability of a locality to recover from damages, decreases in productivity and quality of life impacts due to extreme events, without significant dependence on outside assistance (Mileti, 1999). Additionally, social-ecological resilience can be described as the capacity of socio-ecological systems (SES) to adapt and transform in the face of extreme events, in a manner that continually fosters human well-being (Folke et al., 2016). Collectively, transformations in the social and ecological elements that maintain the structure, function and identity of SES in the face of perturbations, stressors and change are at the core of improving resilience (Moore et al., 2014).

In terms of natural hazard events, resilience characterizations provide a means of identifying risk profiles, degrees of preparedness, and the ability of communities to respond and recover. General resilience strategies as described by Carpenter et al. (2012) are useful for developing scalable resilience assessment approaches. For example, in the case of natural hazard events, general resilience can be characterized in context of multiple exposure types (e.g., hurricanes, droughts, wildfires). General resilience approaches applied to similar geographies with similar hazard profiles provide a means to examine characteristics that contribute to resilience and identify opportunities to improve resilience in a comparable manner. One approach to capture general resilience is composite indices that can be deconstructed into information that may be used to tailor practices to system characteristics (e.g., governance, social aspects, ecological conditions) for resource allocation and implementation of policies and actions to increase resilience. Such approaches can be used as a starting point to address the six objectives identified by Mileti (1999) to sustainably mitigate hazards to reduce catastrophic losses due to natural hazard events nationwide—(1) maintain and enhance environmental quality, (2) maintain and enhance people’s quality of life, (3) foster local resiliency and responsibility, (4) recognize that vibrant local economies are essential, (5) ensure inter- and intra-generational equity, and (6) adopt local consensus building. Geographically-specific resilience characterizations are needed to target the use of local resources to increase community resilience.

**FIGURE 1 |:**
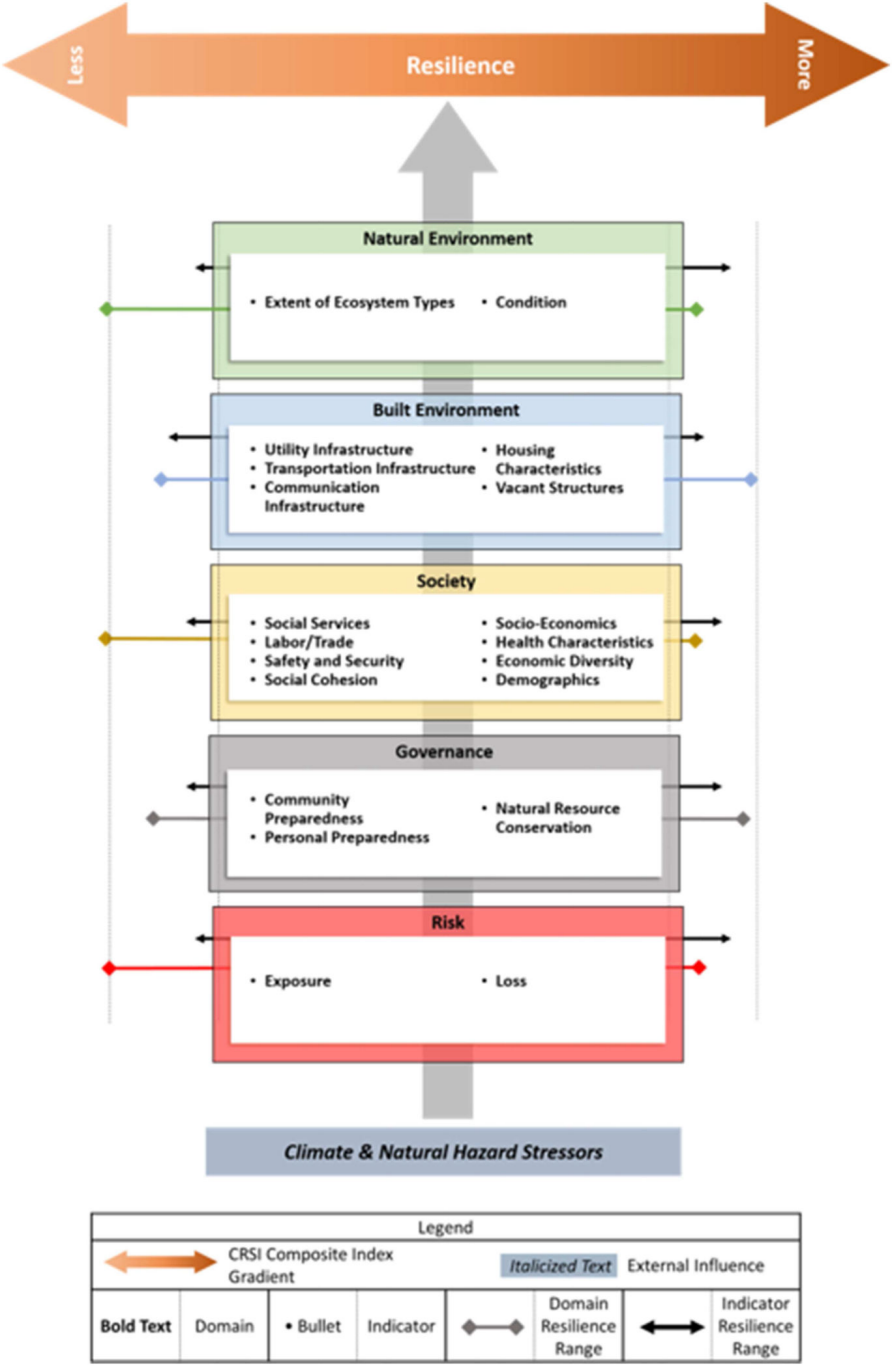
Conceptual representation of the Climate Resilience Screening Index (CRSI) Approach (Summers et al., 2017a).

From a policy perspective, there is a need for nationally consistent measures of community resilience that address the potential widespread socio-ecological impacts of natural hazard events. Summers et al. (2017a) conducted an extensive review of over 500 resilience domains and indicators, and nearly 1,200 metrics associated with the vulnerability and recoverability of social and natural systems in context of natural hazard events (e.g., Pratt et al., 2004; Esty et al., 2005; Hazards and Vulnerability Research Institute (HVRI), 2010; Esnard et al., 2011; Joerin and Shaw, 2011; Meher et al., 2011; ARUP, 2014, 2015; Cutter et al., 2014; Batica, 2015). No singular approach among existing composite measures of natural hazard resilience from the literature was identified to adequately address a combination of multiple hazards exposures, governance, and strengths in social, built and natural environments, for U.S. counties, boroughs and parishes nationwide. However, the reviewed literature provided the building blocks (e.g., individual metrics, suites of indicators and descriptive domains) for developing the new climate resilience screening index (CRSI) as outlined in the Summers et al. (2017b) conceptual framework for constructing the CRSI (Figure 1).

**FIGURE 2 |:**
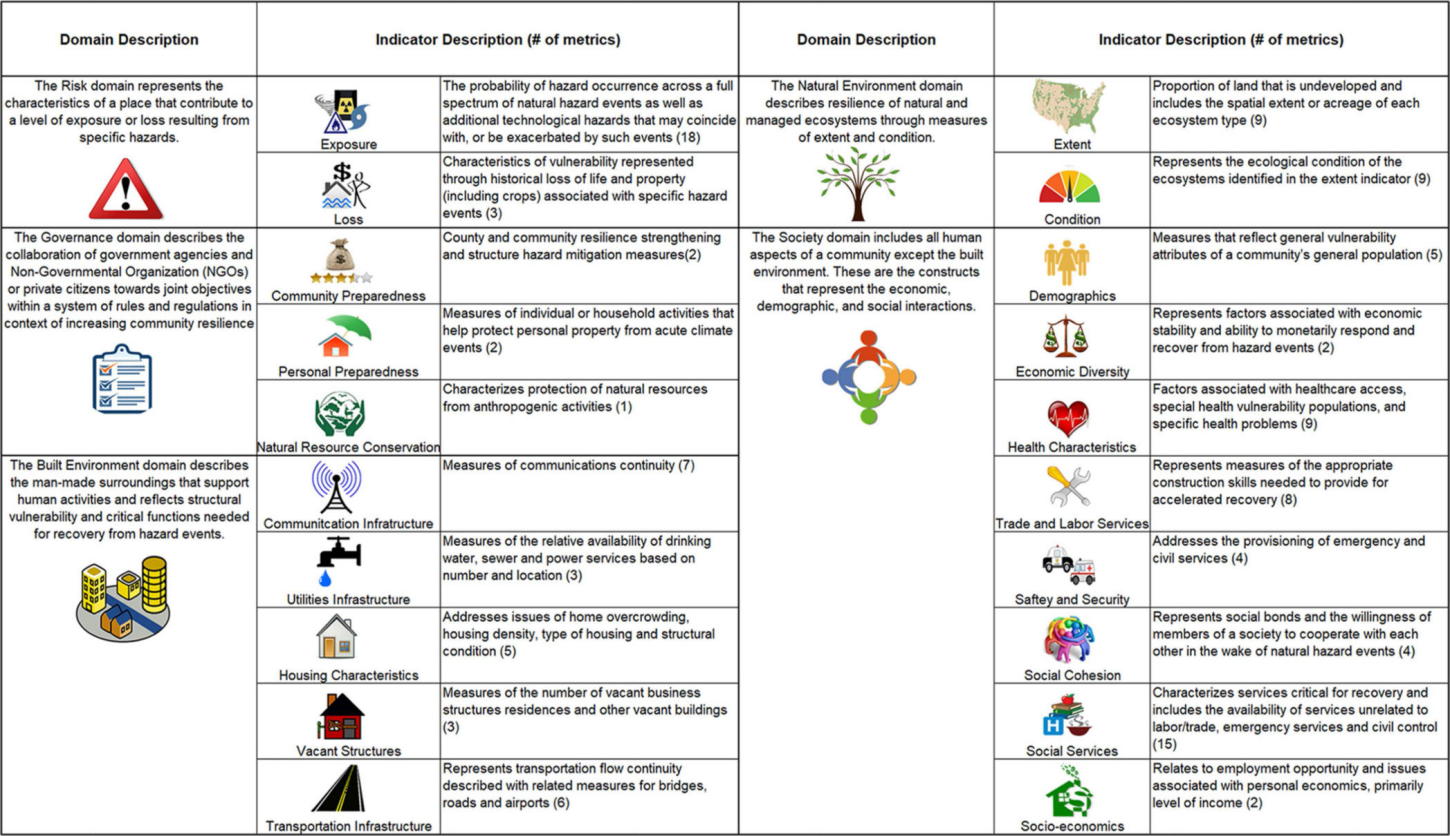
Description of five CRSI domains and twenty indicators; (#) indicates the number of metrics used to calculate each indicator.

The Climate Resilience Screening Index (CRSI) is a composite measure for characterizing the resilience of SES in the context of governance and risk to natural hazard events. CRSI is a composite index, including five domains (Risk, Governance, Society, Built Environment, and Natural Environment) made up of 20 indicators and 117 metrics (Summers et al., 2017a; Figure 2). CRSI was developed for all U.S. counties for the years 2000–2015, with resilience characterizations based on the full range of nationwide county domain scores. CRSI is particularly well-suited as a screening approach, as it characterizes general resilience in a multi-hazard landscape. It does this by addressing twelve types of weather and natural hazard events and eight technological hazards (e.g., contaminated sites and toxic release locations, nuclear facilities and resource conservation and recovery act sites) as part of the exposure indicator of the Risk domain.

The CRSI approach is unique in that it addresses a combination of multiple hazards, governance, and, strengths and weaknesses in social, built and natural environments, for U.S. counties, boroughs and parishes nationwide. The structure of CRSI, the methods used to construct the index and the use of publicly available data lend themselves to a valid approach to rescaling for specific U.S. geographies. CRSI provides a comparative characterization of county-level resilience for all U.S. counties, thus permitting smaller evaluations of regions or states at the same broad comparative level, i.e., inclusive of all counties (Summers et al., 2018). This type of assessment is useful because it measures a broad set of indicators yet reduces resilience to a single unit of measurement while still allowing evaluations at the levels of individual domains and related indicators to permit a deeper understanding of the relationships among resilience concepts to inform management actions (Quinlan et al., 2015).

CRSI characterizes the appropriate components to identify communities based on their hazard profile and other characteristics; however, the individual metrics of CRSI have not been analyzed within specific geographic locations. These geographically specific characterizations are needed to inform local decision making. An example of such a geographic grouping of communities with similar hazard exposures is coastal areas, which have varying degrees of resilience within regions. Coastal areas face specific unique resilience challenges with increased vulnerability to human and natural threats. The multiple threats and impacts natural hazard events, like hurricanes, have raised concerns from policy makers, stakeholders and residents regarding the long-term viability of coastal communities (Reams et al., 2012).

Evidence of the challenges to coastal resilience was demonstrated by two recent events, Hurricane Katrina and the Deepwater Horizon oil spill (Zhang et al., 2015). Coastal counties are an important national asset with tremendous economic value and a substantial proportion of the U.S. population. In 2015, the U.S. ocean and Great Lakes economies accounted for an estimated 128 billion in wages, 320 billion in goods and services, and directly employed 3.2 million people [National Oceanic and Atmospheric Administration (NOAA), 2018]. The U.S. ocean economy accounted for 12 percent more employment than U.S. crop production, telecommunications and building construction industries combined. Many of these employees not only work in, on or near the oceans, but also live nearby. According to the National Coastal Population Report [National Oceanic and Atmospheric Administration (NOAA), 2013], in 2010, 39% of the U.S. population lived in coastal shoreline counties, <10% of the total land area. CRSI can be complemented by additional information such as economic vulnerabilities specific to coastal shoreline areas and societal aspects that may be directly influenced by actions aimed at increasing resilience.

In context of social resilience, diversity of livelihoods may be an important factor in buffering the impacts of natural hazard events (Adger et al., 2005). Location Quotient (LQ) is a way of quantifying how concentrated an industry is in a location compared to a larger geographic area. LQ can be used to examine economic dependencies based on employment data, where higher industry LQ indicates higher reliance on that industry, relative to other locations in the larger geographic area. For coastal and shoreline counties, examining the employment base in ocean economic sectors relative to the total employment in the county may be useful in further characterizing the resilience of these geographic areas. Those regions frequently exposed to hurricanes and coastal flooding, may be even more vulnerable given the direct impact these natural hazards can have on coastal resource-dependent industries. Lower economic diversity and high dependence on the ocean economy combined with lower resilience scores provides additional information regarding employment dependence and lower economic diversity as a potential vulnerability within a region.

The nation-wide CRSI results provide a scale of assessment important for broad national policy development and targeting areas for improvement; however, it does not allow more in-depth assessments for areas that share common traits (e.g., specific risks) or share smaller common boundaries (e.g., state, multiple adjacent counties). Similarly, the unscaled CRSI is a screening assessment and does not evaluate CRSI alongside other tools more specific to economic or quality of life information. To address these issues, CRSI analyses were rescaled to coastal regions and coupled with LQ values to characterize the differences among coastal counties. We differentiate and compare risk components and evaluate relative domain contributions to resilience within each of within each of eight coastal regions. For the Gulf of Mexico (GOM) region, we present CRSI values in conjunction with Location Quotients (LQ) based on ocean economy sector employment for a subset of coastal counties/parishes. The objective is to demonstrate the approach for rescaling CRSI for coastal areas and to present the potential value of a combined index approach to regionally characterize resilience for coastal counties with economic dependence on coastal resources.

## METHODS

The dependence on ocean and Great Lakes economies and the specific set of hazard designations associated with U.S. coastal shoreline counties provides the set of coastal counties upon which CRSI was recalculated to characterize coastal shoreline counties’ resilience within the context of each of eight coastal regions. The re-scaled CRSI scores were then examined in relation to county dependence on the ocean economy as represented by the calculated location quotient (LQ) for the total ocean economy and six ocean sectors. In the GOM region, a subset of coastal counties with ocean economy LQ (LQ_OE) values >1.0 (high dependence on the ocean economy) were also characterized by examining levels of resilience (CRSI scores) along with calculated LQ values.

### Defining Coastal Counties

The coastal and coastal shoreline counties identified for regional scaling of CRSI were selected from NOAA’s Economics: National Ocean Watch (ENOW) 2015 dataset^1^. The ENOW 2015 dataset includes 402 coastal shoreline and coastal counties in 30 coastal states in eight regions. The coastal shoreline counties in this data set are based on the Federal Emergency Management Agency’s designation requiring a coastal county to have a coastline bordering the open ocean or the Great Lakes, or contain coastal high hazard areas referred to as V-zones, special flood hazard areas or areas that are vulnerable to high velocity wave action from storms or seismic activity)^2^. The ENOW dataset excludes shore-adjacent counties with no relevant economic activity (11 counties and the District of Columbia) and includes 17 additional counties that are not shore-adjacent but do have significant ocean and Great Lakes economic dependence. Of the 402 counties in the ENOW dataset, CRSI metric data were available for 398 counties. Hereafter, these 398 counties will be referred to as ENOW coastal counties. Each county was assigned to an ENOW designated region to rescale CRSI for regional characterizations.

The number of counties within each region are as follows:

Great Lakes (85)

Gulf of Mexico (68)

Mid-Atlantic (93)

North Pacific (19)

Northeast (28)

Pacific (5)

Southeast (53)

West (47).

### Rescaling CRSI Within All ENOW Counties and Within Each Coastal Region

Original CRSI resilience characterizations were for all U.S. counties for the years 2000–2015, scaled based on the full range of nationwide county scores. These national scale resilience characterizations are intended to provide information and results comparable across all U.S. counties and regions. We rescaled CRSI using the ENOW coastal counties and regions as identified above. The ENOW regional rescale for CRSI was done by limiting the range of metric values assessed to only those for counties within each of coastal region. ENOW coastal county CRSI results for the coastal regions are comparable only within each region. Re-scaled metric values were used to calculate indicator and domain scores following Summers et al. (2018).

The Risk domain includes two indicators, exposure and loss. Metrics were derived as a probabilistic calculation based on geophysical and technological hazards as described in Buck et al. (2018). Natural hazards exposures characterized include hurricanes, tornadoes, inland flooding, coastal flooding, earthquakes, wildfires, drought, high winds, hail, landslides, extreme low temperatures, and extreme high temperatures. Human and monetary losses were quantified and attributed to each hazard. The product of the exposure and loss scores for each county resulted in the county Risk domain value, which contributed to the final CRSI calculation.

We calculated domain scores for each of the other four domains (Governance, Society, Built Environment and Natural Environment) from re-scaled metric scores. Summers et al. (2018) calculated metric values from data that were adjusted for age, population or spatial area, as appropriate, prior to standardization (e.g., number of hospitals in a county adjusted by the population of the county). We utilized the adjusted metric values in our re-scaling to calculate metric scores. The scores for each of the four domains were calculated in the following manner:

Metricscore=Min-maxstandardizedmetricvalues

Indicatorscore=Min-maxstandardizedsumofmetricscores

Domainscore=Min-maxstandardizedsumofindicatorscores

All domains for each ENOW coastal county were min-max standardized on a scale from 0.01 to 0.99. The final CRSI calculation begins as a scaled value for recoverability/vulnerability derived from Governance and Risk (basic CRSI) with the Governance value being adjusted by the remaining domain scores for social, built environment and natural environment to complete the calculation of CRSI as shown below:

$$CRSI_{\text{i}}=\left(Gov_{\text{i}}+Soc{\left(a\right)}_{\text{i}}Gov_{\text{i}}+BE{\left(a\right)}_{\text{i}}Gov_{\text{i}}+NE{\left(a\right)}_{\text{i}}Gov_{\text{i}}\right)/Risk_{\text{i}}$$

where CRSI_i_ = the value of CRSI or adjusted resilience for county i and Soc(a)_i_, BE(a)_i_, and NE(a)_i_ are the adjustment multipliers for Society, Built Environment, and Natural Environment in each county i, and Risk_i_ is the Risk score for county i. The adjust factors are calculated as:

$$Soc{\left(a\right)}_{\text{i}}=\left(Soc_{\text{i}}-Soc_{\text{m}}\right)/Soc_{\text{m}}$$

where Soc(a)i is the adjustment multiplier for society in county i, Soc_i_ is the social domain score for county i and Soc_m_ is the median social domain score for all counties;

$$BE{\left(a\right)}_{\text{i}}=\left(BE_{\text{i}}-BE_{\text{m}}\right)/BE_{\text{m}}$$

where BE(a)_I_ is the adjustment multiplier for built environment in county i, BE_i_ is the built environment domain score for county i and BE_m_ is the median built environment domain score for all counties;

$$NE{\left(a\right)}_{\text{i}}=\left(NE_{\text{i}}-NE_{\text{m}}\right)/NE_{\text{m}}$$

and where NE(a)_I_ is the adjustment multiplier for natural environment in county i, NE_i_ is the natural environment domain score for county I and NE_m_ is the median natural environment domain score for all counties.

The distribution of ENOW coastal county CRSI scores within each coastal region was examined to identify domain level differences in county resilience characterizations and to compare natural hazards profiles. Regional coastal re-scale CRSI results were examined to look at top and bottom-ranked counties and to highlight domains positively and negatively influencing overall scores. CRSI scores for the GOM region were then supplemented with location quotients for the total ocean economy and ocean sectors to characterize potential socio-economic vulnerabilities specific to ENOW coastal counties.

### Location Quotients for Total Ocean Economy and Ocean Sectors

The CRSI Society domain includes an Economic Diversity indicator calculated from two metrics, the Gini coefficient and The Hachman Index (Summers et al., 2017b). The Gini coefficient represents the distribution of wealth and is the most commonly used measurement of inequality. The Hachman Index is the reciprocal of the sum of location quotients for 2 digit NAICS codes weighted by industry shares. Hachman Index values show how similar an area is to another larger area (i.e., for CRSI county to nation) with values bound between 0 and 1.0. A value of 1.0 indicates identical industry employment distributions between the area of interest and the reference area (high diversity). A Hachman Index value of 0.0 represent completely dissimilar industry concentrations (lowest diversity). The broader Hachman Index used in CRSI may not reveal economic vulnerabilities that are particular to specific geographies like ENOW coastal counties.

NOAA’s Economics: National Ocean Watch (ENOW) data includes employment data statistics derived from the Bureau of Labor Statistics’ Quarterly Census of Employment and Wages data. ENOW provides both employment data for Total Ocean Economy, and for the six ocean sectors it includes^3^.

Total Ocean Economy (OE)- All ocean economic activities within a geography.

1. Living Resources (LR)- Commercial fishing, fish hatcheries, aquaculture, seafood processing, and seafood markets; recreational fishing is excluded (included in Tourism and Recreation).
2. Marine Construction (MC)- Beach nourishment and harbor dredging.
3. Ship and Boat Building (SBB)- Ship and boat building and repairs.
4. Marine Transportation (MT)- Deep sea freight, marine passenger transportation, pipeline transportation, marine transportation services, search and navigation equipment, and warehousing.
5. Offshore Mineral Extraction (OME)- Oil and gas exploration and production, and sand and gravel mining.
6. Tourism and Recreation (TR)- Eating and drinking establishments, hotels, marinas, boat dealers and charters, campsites and RV parks, scenic water tours, manufacture of sporting goods, amusement and recreation services, recreational fishing, zoos, and aquariums.

Each of these six ocean sectors has specific North American Industry Classification System (NAICS) industry codes associated with it4z^4^ County total ocean economy employment data and employment data for the six ocean sectors were obtained from NOAA’s ENOW API^5^.

An ocean economy location quotient (LQ_OE) for each of the ENOW coastal counties in the GOM Region was calculated based on the proportion of county employment in the ocean sector to the total employment in the county^6^ as a proportion of the ratio of the total employment in the ocean sector within the coastal region to the total employment in all ENOW coastal counties. In the calculation of each ocean sector LQ, the concentration of employment in each sector was compared to the concentration of that sector employment for all ENOW coastal counties within that region in context of total ocean economy employment for the region.

Location quotients for the ocean economy and the six sectors were calculated for each GOM ENOW coastal county as follows:

$$LQ_{i}=\left(e_{i}/e\right)/\left(E_{i}/E\right)$$

Where,

LQ = location quotient for the total ocean economy or sector (i); e_i_ = county employment in the total ocean economy or ocean sector, e = total county employment for ocean economy LQ calculation or total county employment in ocean economy for ocean sector LQ calculations; E_i_ = GOM region employment in the ocean economy or sector; E = total GOM region employment for ocean economy LQ calculation or total GOM region employment in ocean economy for ocean sector LQ calculations.

### Examining CRSI and LQ Values

A subset of counties (*n* = 42) within the GOM region with LQ_OE values > 1.0 were selected to examine employment dependence in context of resilience scores. First, all values were percentile ranked. Each county CRSI score and LQ_OE value was categorized using the median for each measure, as estimated from the subset of regional values. Counties with LQ_OE and CRSI values below the GOM regional median value were categorized as having lower ocean economic dependence and lower resilience. Counties with LQ_OE and CRSI values above the median for the regional subset were categorized as having higher ocean economic dependence and higher resilience. LQ_OE and CRSI percentile ranked values were plotted as a four-quadrant bubble plot displaying the degree of resilience and dependence on the ocean economy for a visual comparison.

## RESULTS

### CRSI for ENOW Coastal Counties: Regional Coastal Rescale

**FIGURE 3 |:**
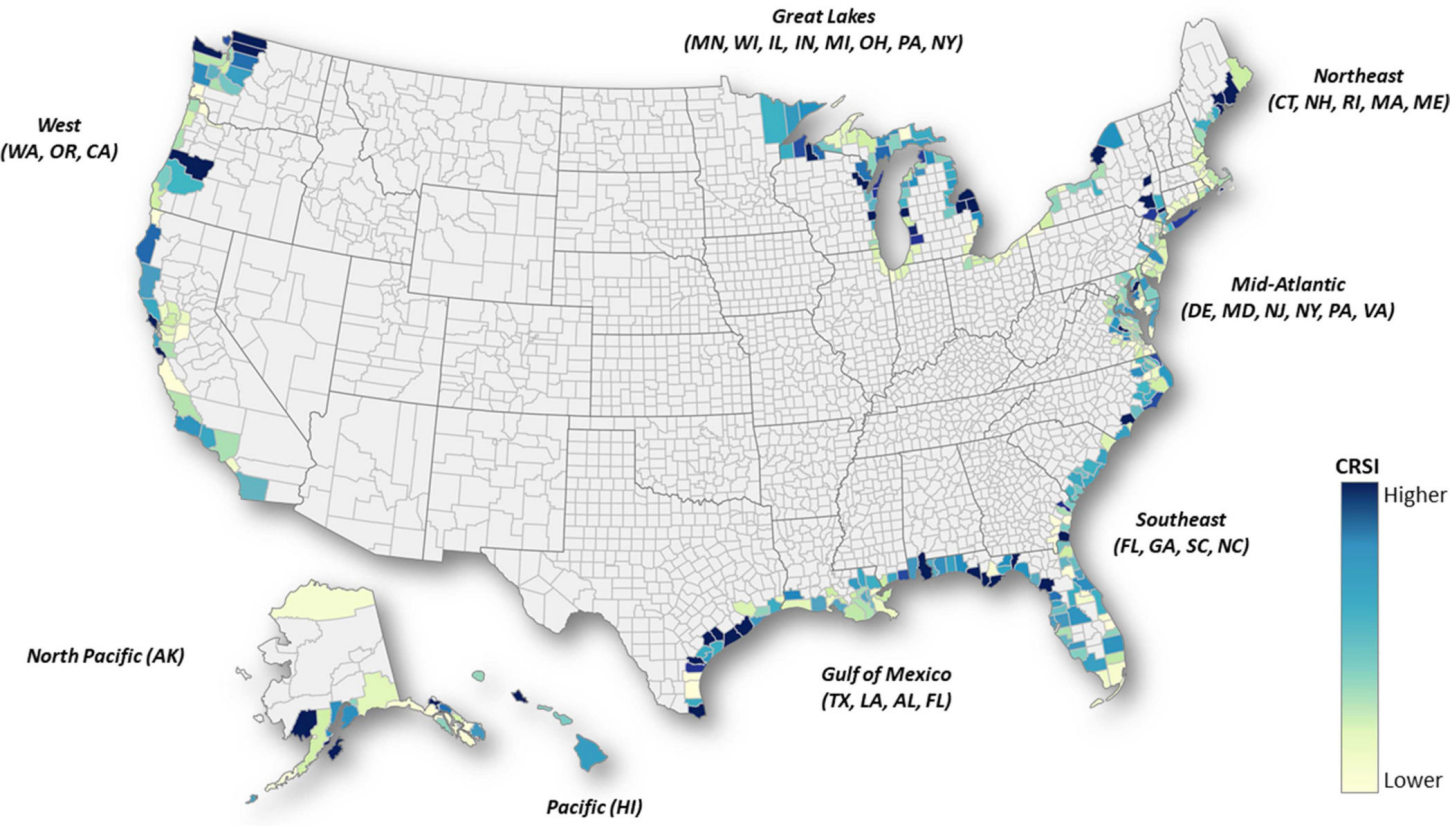
Distribution of CRSI scores for ENOW counties within each of the eight coastal regions. Lighter colors represent lower scores (less resilience) and darker colors, higher scores (more resilience). Color scale is based on percentiles associated with CRSI values within each region.

The map shows the distribution of CRSI scores scaled within each of the eight coastal regions (Figure 3). The color scale is based on the range of county, borough and parish CRSI values within each of the eight regions. The range of CRSI scores within each region are represented in Table 1. Scoring metrics within the regions results in indicator, domain and CRSI scores much different than those resulting from national assessments. Since the range of metric values used to set the minimum and maximum values used to score metrics is set within each region, CRSI scores re-scaled within each region are comparable only within the region not across regions. The regional re-scaled CRSI results now show higher and lower scores distributed throughout each coastal region, a resolution among scores that is otherwise diluted in the original national assessment and is more useful for targeting resources within these regions to increase resilience to a shared set of natural hazards. The CRSI scores and the accompanying domain scores resulting from the coastal regional rescale are included in the Supplemental Material (ENOW regional re-scaled results sheet).

Rescaling CRSI within coastal regions puts ENOW coastal counties in context of similar natural hazard exposures, further highlighting sub-regional county differences. Re-examining ENOW coastal counties within coastal regions (Figure 3; Table 1) demonstrates an enhanced range of resilience scores for the Northeast, Mid-Atlantic and GOM regions. This spread of CRSI scores and component domain scores makes it easier to discern potential county resilience related strengths and weaknesses. An ENOW coastal county may appear to have a “good” score for a domain on a national scale while being below the median for the re-scaled regional score for the same domain. For example, when scaled within coastal regions, the ENOW counties Ulster County, NY and Victoria County, TX have high CRSI scores (>100) and Wayne County, GA and Kenedy County, TX have low CRSI scores (< −20). In the Gulf Region, Victoria County, TX ranked high among all ENOW counties in the original CRSI (2nd) and ranked 1st in the ENOW regional re-scale. Kennedy County, TX CRSI scores dropped in rank from 59th in the national CRSI to 68th in the ENOW regional re-scale [Rankings within each region are included in the Supplemental Material for the original national and ENOW regional re-scaled results along with the change in rank due to the re-scaling efforts (Rescale Change in Reg rank sheet)].

Counties with the highest and lowest CRSI scores (top and bottom 5) are presented for each regional characterization (Table 1). The top and bottom-ranked CRSI scores within each region is provided to illustrate how differences in the domain component scores can contribute to higher and lower CRSI scores. Since the scores for each domain scaled within region, the number represents a position on the scale from 0.1 to 0.9 (lowest to highest) for that region. Counties with the lowest CRSI scores may score higher in some domains than those counties with the highest CRSI scores in the region, but overall, it is the combination of higher risk, lower Governance scores and the median adjusted scores for Society and the Natural and Built Environment domains (not the domain scores shown in Table 1) that determine the CRSI score (see CRSI equation in Methods section). A summary follows for each region’s top and bottom ranking counties, as re-scaled county values are only comparable within region.

### Great Lakes

Among the top-ranked CRSI scores in the counties of the Great Lakes region, risk domain scores were low (less risk), contributing to higher CRSI scores. For the lowest CRSI scores in the region, Risk domain scores were high and most counties ranked in the bottom five had lower scores in the Society domain. The highest and lowest Governance domain scores within the entire region were in Keweenaw County, MI and Erie County, PA respectively; both ranked in the bottom five for CRSI scores in the region. Keweenaw County, MI also had the lowest Society domain score for the entire region. Built Environment domain scores varied across the top and bottom-ranked counties. The majority of Natural Environment domain scores among the top and bottom-ranked counties fell within the upper 50th percentile range for the region.

### Gulf of Mexico

Governance domain scores were generally in the upper 50th percentile with the exceptions of Liberty, Gulf and Monroe counties in Florida. Monroe, Florida ranked last in the Governance domain among all GOM counties. Risk domain scores were lowest for the highest-ranking counties for CRSI, except Brazoria, TX, which had a higher Risk domain score than the other top five and bottom five counties except Orleans, LA. All ranked counties shown had Risk domain scores in the lower 25th percentile across the GOM region. Of the top and bottom ranked counties, only three had scores above the 50th percentile for the Built Environment domain. Counties with the lowest CRSI scores also scored low in the Society domain except Monroe County, Florida. Natural Environment domain scores for the top and bottom-ranked counties in the GOM were generally lower for the bottom-ranked counties except for Liberty County, FL.

### Mid-Atlantic

As seen within many of the regions, Risk domain scores were lowest among the top-ranking counties in the Mid-Atlantic region. However, both Salem County, NJ and Somerset County, MD scored low for Risk and scored among the bottom five ranked CRSI scores. Scores for the Built Environment, Society and Natural Environment domains were much lower for the bottom-ranked counties except Salem County, NJ and Somerset County, MD which scored highest among the ranked counties shown for the Natural Environment domain. Arlington County, VA ranked scored the lowest in the region for the Natural Environment domain. King William County, VA scored low for the Built Environment domain among the top five CRSI ranked counties. Governance domain scores shown for the ranked counties were all in upper 50th percentile for the region except Baltimore City, MD and Arlington County, VA.

### North Pacific

In the North Pacific region (Alaska), Kodiak Island Borough scored highest for CRSI and Bristol Bay Borough scored lowest. Risk domain scores among the highest and lowest ranked counties for CRSI were low except for Yakutat City Borough which scored the much higher (0.56). Governance domain scores varied across the counties shown with Bristol Bay Borough scoring the highest among the top and bottom-ranked counties and Wrangell City Borough scoring the lowest in the North Pacific region. These same two boroughs scored lowest in the region for the Built Environment domain. Society domain scores also varied across the top and bottom-ranked boroughs; however lower scores are shown for the Census Areas of Dillingham and Hoonah-Angoon and for the boroughs of Bristol Bay and Wrangell City. Natural Environment domain scores shown were all greater than the 50th percentile for the region with the Dillingham Census Area scoring highest within the North Pacific region.

### Northeast

Hancock County, ME scored highest for CRSI in the Northeast region, while Nantucket County, MA scored lowest. Risk domain scores were lowest among the top-ranked CRSI counties in the Northeast region except Lincoln County, ME. Built Environment domain scores varied across the ranked counties shown with one of the top-ranked counties, Lincoln County, ME scoring low and Bristol, RI scored the lowest for this domain within the entire region. The lowest score within the region for the Governance domain was also shown for Bristol County, RI and the score was also low for New London County, CT. New London County also had the lowest Society domain score for the Northeast region; Lincoln County, ME the highest. All but two ranked counties, Strafford County, NH and Suffolk County, MA, scored above the regional mean for the Natural Environment domain.

### Pacific

The highest Risk domain scores among the five counties of the Pacific region (HI) are shown for the two top CRSI ranked counties, Honolulu and Hawaii. Hawaii County had the highest Risk domain score for the region, but also scored highest for the Built Environment domain. Hawaii County scored highest in the region for the Society and Natural Environment domains. Maui scored low in the Natural Environment domain, as did Kauai County. Kauai County also scored lowest in the region for the Built Environment domain. Kalawao County scored lowest in this region for the domains of Governance, Society and the Natural Environment and also scored low in the Built Environment domain.

### Southeast

Within the Southeast region, Pender County, NC scored highest for CRSI and Wayne County in GA scored lowest. All but two counties scored above the median for the Governance domain. Risk domain scores for the top and bottom-ranked counties in the region were all low. Only one ranked county shown, Pender County, NC, scored in the upper 50th percentile for the Built Environment domain. Lower Built Environment scores are shown for the bottom-ranked counties. Brantley County, GA scored lowest in the region for the Society domain and all bottom-ranked counties in the region scored lower for this domain. Similarly, Natural Environment domain scores for the lower ranked counties were all lower than those with the highest CRSI scores.

### West

The highest and lowest CRSI scores for the West region were in Clallam and Wahklakum Counties in WA, respectively. Higher Governance domain scores are shown for the counties that ranked lowest for CRSI in this region. Counties ranked in the bottom 5 for CRSI had higher Risk domain scores. Lower scores for the Built Environment, Society and Natural Environment domains are shown for most of the bottom-ranked counties with the exception of San Joaquin County, CA, which scored higher for the Built Environment domain and Del Norte County, CA which scored higher in the Natural Environment domain.

Resilience characterizations within geographically, economically and socially similar areas may foster peer to peer transfer of best practices. For example, in the GOM region, Kenedy County, TX (CRSI = −22.2) might look to best practices in Victoria County, TX (CRSI = 107.8) for built environment, society, and natural environment activities to improve Kenedy County resilience. These two GOM counties have similar risk profiles. Similarly, in the Mid-Atlantic region, Somerset County, MD (CRSI = −1.75) and King William County, VA (CRSI = 23.89) have similar risk profiles but Somerset has significantly lower domain scores in all categories. King William County could provide Somerset with examples of actions that have enhanced King William’s governance, built and natural environments or social systems.

### Ocean Economy Employment Dependence in the GOM Region

The location quotients >1.0 for the total ocean economy (LQ_OE) and the corresponding ocean sector location quotients for GOM counties are included along with the CRSI scores (Table 2). Forty two counties/parishes in the GOM region had total ocean economy LQ values (LQ_OE) >1.0, indicating a higher dependence on the ocean economy compared to all ENOW counties in the GOM region. Monroe County, FL and Plaquemines Parish, LA both ranked in the top ten for LQ_OE values and both rank in the bottom 10 for CRSI scores among the ocean economy dependent counties and parishes in the region. However, for both Monroe County and Plaquemines Parish the ocean economy is diverse with tourism and recreation being a major component in Monroe County and employment concentrated heavily in offshore mining and extraction (OME) and in marine construction (MC) in Plaquemines Parish. Monroe County ranks 4th from last in the region for CRSI with a high score for the Society domain which includes economic diversity. The information in Table 2 can be used to compare counties with ocean economies concentrated in similar sectors. For example Mobile County, AL and St. Mary Parish, LA both have employment concentrated in the ship and boat building industry (SBB) and have similar lower CRSI scores.

**FIGURE 4 |:**
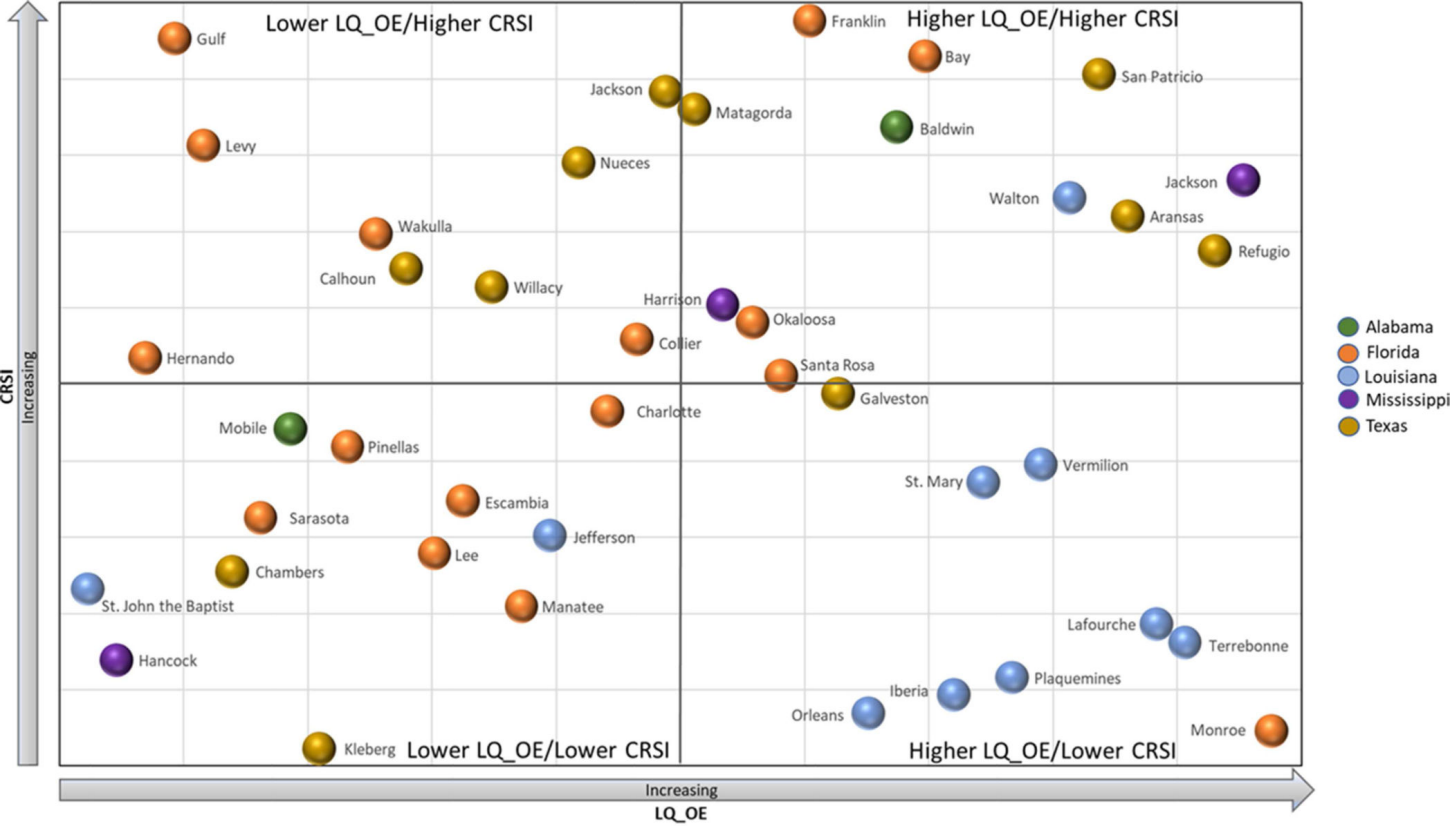
Scatter plot showing LQ_OE (x-axis) and CRSI (y-axis) percentile ranked values for the forty-two GOM coastal shoreline counties with LQ_OE values >1.0. Quadrants are delineated by the median values along the x and y axes. Bubbles are color coded by state.

Percentile ranked LQ_OE, and CRSI scores for the forty-two ENOW coastal counties and parishes in the GOM region are plotted in Figure 4. Median values for LQ_OE and CRSI delineate quadrants along the X and Y axes, respectively. LQ_OE values increase from left to right; CRSI values increase from bottom to top. Each quadrant is labeled accordingly. The color of each bubble represents the state in which the county is located. All counties shown have a dependence on the ocean economy with an employment concentration in ocean sectors higher than that observed across the GOM region, suggesting an added vulnerability in the socio-ecological context of resilience.

As presented, counties falling in the lower right-hand quadrant should be considered more vulnerable compared to the other counties shown in the region. The counties in the upper left-hand quadrant (Lower LQ_OE/Higher CRSI) should be considered less vulnerable. Those counties in the lower right quadrant may be more vulnerable in the GOM region. This quadrant includes four Louisiana parishes with the lowest CRSI scores in the region: Orleans, Iberia, Terrebonne and Lafourche. These parishes also have higher Risk scores. Particular attention needs to be given to the Society domain for these counties, which negatively impacted CRSI scores in the lowest ranked counties for the region, in addition to lower scores in the other domains. All nine parishes in Louisiana had CRSI values lower than most other ocean economic dependent counties.

## DISCUSSION

Coastal counties share certain governance approaches, natural resource practices, built environment requirements, and demographic characteristics not shared with non-coastal counties. Thus, an analysis that includes all counties tends to “dilute” differences among coastal counties. To differentiate among coastal counties with ocean economies, a regional ENOW coastal county rescaled analysis specifies the differences in CRSI scores within the identified region. This rescale differentiates patterns among ENOW coastal counties and allows for a more relevant assessment of potential areas for improvement in coastal areas.

While the original CRSI quantitative resilience scores are useful for comparisons across the nation, they may offer little to direct investment for the enhancement of overall resilience at regional and state levels (Frazier et al., 2013). Applications of the Conjoint Community Resilience Assessment Measurement (Cohen et al., 2013) and the Spatially Explicit Resilience-Vulnerability (SERV) model (Frazier et al., 2014) demonstrate the need for specific local information to address local resilience issues. Re-scaling ENOW coastal county assessments to specific regions provides information regions and states can use to equitably allocate resources for improving infrastructure, protecting and restoring natural environments, and enhancing quality of life in coastal areas. In counties where risk profiles and governance are similar, it is important to take a look at the resilience characteristics (indicators and metrics) identified in CRSI for the Built Environment, Society and Natural Environment domains. It is at this level that states may be able to evaluate differences and allocate resources to support economic vitality, increase resilience and improve overall human well-being for these natural hazard-prone areas (Cutter et al., 2014).

Resilience and sustainability are inextricably linked to economic conditions and activities (Espiner et al., 2017). The Location Quotient for the total ocean economy and each ocean-dependent sector based 6 digit NAICS codesmay reveal economic vulnerabilities overlooked in the broader Hachman Index used in CRSI. Re-scaled CRSI scores account for within-region differences in the economic diversity, but providing location quotient information in addition to the CRSI indicator scores for Economic Diversity highlights an economic vulnerability specific to the GOM region. Similarly, economic scaling can be an issue when examining the relationship between community resilience and long-term tourism (Bec et al., 2016). There is recent consensus among researchers that coastal vulnerability is geographically dependent and requires placed-based information (Bevacqua et al., 2018). The present re-scaling of CRSI is especially important for the GOM region as it is frequently exposed to natural hazards (e.g., hurricanes and coastal flooding) that can directly impact the ocean sector industries upon which these coastal counties depend. The utility of more detailed economic information is increased within the specified coastal regions. At the regional scale, the additional economic measures complement the minimal economic metrics of CRSI resulting in more explicit differentiation and allowing further subgroupings (e.g., targeting areas that concentrate on ship and boat building or tourism). A re-assessment of the GOM region that includes employment dependencies through a location quotient analysis, in addition to regionalized CRSI results, offers a unique characterization of general resilience to natural hazard events that CRSI and LQ do not capture independently (e.g., Morrisey, 2016; Jahn, 2017; Fracasso and Marzetti, 2018). The joint assessment also determines if certain types of economic activities are more vulnerable to certain events.

At a macroeconomic level, community resilience can be clearly influenced by economic sectors (Rose and Krausmann, 2013). Using economic assets to improve housing, infrastructure or employment has been demonstrated to aid decision-making and enhance disaster resilience for coastal communities in the GOM (Cutter et al., 2014) and coastal Oregon (Fischer, 2018). Although connections between community resilience, its capacity level and economic factors have been theorized or demonstrated (Tobin, 1999; Cutter et al., 2003; Buikstra et al., 2010; Cui et al., 2016), some studies have demonstrated no association between economic factors and community resilience for the oil and gas industry in the GOM (Reams et al., 2012) or level of community development in general in the northern GOM (Lam et al., 2016). Natural and anthropogenic hazards (e.g., hurricanes, sea level rise, salt water intrusion and oil spills) in the GOM can pose unique problems to the dominant sources of coastal economies (Kim and Marcouiller, 2015; Zhang et al., 2015; Jurjonas and Seekamp, 2018). Our results suggest employment dependence on ocean sectors and lack of diversity across ocean sectors may be a vulnerability that could potentially reduce coastal community resilience. Particular interest should be focused on those industries that suffer long term effects due to the impacts of natural disasters. The CRSI/LQ combination offers a multi-dimensional approach to examine general resilience characteristics across five domains while identifying potential employment vulnerabilities based on ocean economy dependence and lack of diversity across sectors.

High ocean dependence (LQ_OC) and a lack of ocean sector diversity should be considered an indicator of added economic vulnerability along with the economic indicators already in the CRSI Society domain. For example, parishes in Lousiana with a dependence on Ship Boat Building (SBB) or the fishing industry, tend to have lower resilience scores. These two industries may exacerbate vulnerabilties not pinpointed specifically in CRSI scores. Hurricanes can completely close fisheries for extended periods of time and the impact of coastal flooding and hurricanes may exacerbate contamination exposures (Superfund, RCRA and TRI sites) associated with the SBB industry and other ocean sector industries in addition to directly impacting the economic vitality. It is not suggested that ENOW coastal counties in the GOM region are less resilient overall based on their dependence on the ocean economy, but they may be slower to recover in the event of hurricanes and coastal flooding because of this dependence. Futhermore, loss of employment in ocean economy sectors following natural hazard events could impact the well-being of those coastal communities.

LQ and CRSI information may be used to look within each state to evaluate resilience differences by focusing on the commonality of ocean economic dependence. In Alabama, Baldwin County is two times more dependent on the ocean economy than Mobile County; however, Baldwin County scores much higher for resilience. The counties also have different industry profiles for the ocean economy sector employment. With similar risk profiles, domain scores need to be dissected to the indicator level to evaluate how these economic dependences may influence resilience. Measures such as the LQ could be linked back to a variety of socio-ecological measures used in the CRSI Society domain and is worthy of further examination.

Examining the intersections among different domains and indices is an important aspect of improving SES resilience characterizations (Faulkner et al., 2018). Bringing together elements of resilience and economic information can better inform disaster risk management and foster community organization (Faulkner et al., 2018). Typologies, like the one here using CRSI and LQ, for characterizing ENOW coastal counties in a region of the United States could assist government in developing guidance (for risk, vulnerability, or recovery) that are more applicable to specific local contexts (Chang et al., 2018). Similarly, these groupings can facilitate the development of peer localities networks (in this case coastal counties in the Gulf of Mexico region) for advocacy and knowledge exchange. These rescaled assessments provide within state comparisons that could help states target resources and improve regional resilience considering all counties within a region. Increasing resilience in coastal areas is expected to have a positive influence on human well-being over time. Including more specific quality of life measures with economic information and resilience as defined by CRSI would further enhance a locality’s ability to learn from and respond to extreme weather events.

Regionally-derived resilience indices for GOM counties (Lam et al., 2016) have shown “ranking” patterns similar to our GOM re-scaled regional CRSI analysis. However, differences in scale, selection of metrics and analytical methods can all yield different results. For example, our results differ from national assessments like Baseline Resilience Indicators for Communities (BRIC; Cutter et al., 2014). BRIC results show Louisiana parishes among the most resilient counties in the U.S, while regional ENOW coastal county assessments rank them among the lowest. Results of CRSI should be further compared (fidelity tested) to additional resilience assessment results. Such comparisons to the CRSI approach and results may be useful to refine or complement existing measures. For example, our data-driven typological approach would be useful in conjunction with Sempier et al. (2010) Coastal Community Resilience Index, an internal self-assessment tool, developed to help communities address resilience issues and identify how to allocate resources to prepare and recover from disasters. Although the county-level of assessment CRSI provides is at a smaller scale than many other assessments, e.g., island-nation, country, region (Arkema et al., 2013; Selig et al., 2018), some community level tools and decisions require more localized scales. Future work could explore down-scaling CRSI results to sub-county scales to inform these types of tools and decisions.

The CRSI re-scaling approaches described in this paper can be transferred to other specific geographies and the data used to calculate CRSI and LQ values are both publicly available. Employment dependence as presented for LQ can be tailored to specific industries within the geographic area of interest. However, approaches to refine rescaled CRSI characterizations for geographically specific areas may be needed and could include a finer scale population-based exposure characterization of risk. Additionally, metrics for the Governance domain of CRSI could be further developed for specific geographic areas using regional or state level resilience plans not consistent enough for national comparisons. In coastal areas, the number of ecological restoration, structural protection, and/or non-structural risk reduction projects that are planned or being implemented may be appropriate to improve natural or built environmental domain measures. For example, Louisiana’s Comprehensive Master Plan for a Sustainable Coast (Coastal Protection Restoration Authority of Louisiana, 2017) identifies restoration projects to build or maintain land and support productive habitats for commercially and recreationally important activities, structural protection projects to reduce flood risk by acting as physical barriers against storm surge and non-structural risk reduction projects to elevate and floodproof buildings and help property owners prepare for flooding or move out of high-risk areas. The natural environment component of CRSI could be potentially linked to community-based natural resource management strategies to strengthen SES resilience assessments for coastal areas (Delgado-Serrano et al., 2018).

Another consideration for refining CRSI for regional applications may be a substitution of regional, more specific LQ values for the Hackman Index as the sole measure of economic diversity, and inclusion of the Gini coefficient as a metric of socio-economics. Lastly, the addition of regionalized quality of life measures to the CRSI/LQ typology would be useful for setting baselines to help communities look to one another for improving their most important quality of life aspects and to better track progress over time following extreme weather events and natural disasters. These combinations of measures and further refinements will strengthen U.S. coastal county resilience characterizations and may increase the likelihood of improving environmental, social and economic recovery outcomes.

## CONCLUDING REMARKS

ENOW coastal counties share certain governance approaches, natural resource practices, built environment requirements, and demographic characteristics. These qualities support the need for resilience characterizations specific to coastal regions. Although national level assessments exist, re-scaling CRSI assessments to ENOW coastal counties within regions highlights resilience strength and weaknesses otherwise diluted in the national level assessments. In addition, combining the LQ as measure of employment dependence on ocean economies with CRSI scores captures a potential vulnerability specific to coastal counties which is not reflected in CRSI alone. Including regionalized quality of life indicators/indices to the CRSI/LQ typology could help strengthen resilience assessments as an additional measure of vulnerability of the people living and working in ENOW coastal counties. The information presented can help communities identify opportunities (i.e., targeting resources, knowledge transfer) to strengthen resilience and improve overall community well-being. The unique approach of combining CRSI and LQ for characterizing natural hazard resilience described could be transferred to other specific geographies as defined by population groups, hazard profiles and economic dependence.

## Supplementary Material

SI

## Figures and Tables

**TABLE 1 |:** Highest and lowest CRSI scores within each of the coastal regions.

Region	Rank	County, State	CRSI	GOV	RISK	BLT	SOC	NAT
Great Lakes	1	Oceana, MI	24.66	0.62	0.01	0.38	0.39	0.41
	2	Huron, MI	13.91	0.93	0.08	0.53	0.66	0.33
	3	Ashland, WI	12.67	0.89	0.13	0.25	0.83	0.86
	4	Sheboygan, WI	11.87	0.59	0.10	0.51	0.74	0.73
	5	Jefferson, NY	11.30	0.66	0.09	0.74	0.39	0.66
	81	Erie, PA	0.06	0.01	0.24	0.65	0.43	0.58
	82	Luce, MI	−0.05	0.80	0.38	0.07	0.25	0.76
	83	Wayne, MI	−0.22	0.52	0.48	0.63	0.14	0.07
	84	Lucas, Ohio	−0.31	0.37	0.47	0.35	0.36	0.01
	85	Keweenaw, MI	−1.22	0.99	0.44	0.21	0.01	0.60
Gulf of Mexico	1	Victoria, TX	107.83	0.54	0.01	0.54	0.83	0.57
	2	Jefferson, FL	75.46	0.81	0.01	0.16	0.87	0.44
	3	Franklin, FL	64.49	0.52	0.01	0.08	0.98	0.82
	4	Gulf, FL	25.60	0.26	0.01	0.15	0.53	0.74
	5	Brazoria, TX	14.71	0.82	0.18	0.89	0.72	0.81
	64	Orleans, LA	0.35	0.71	0.24	0.29	0.41	0.32
	65	Monroe, FL	0.35	0.01	0.06	0.58	0.82	0.53
	66	Liberty, FL	0.20	0.43	0.07	0.05	0.01	0.85
	67	Kleberg, TX	−0.35	0.66	0.03	0.21	0.29	0.43
	68	Kenedy, TX	−22.20	0.85	0.02	0.27	0.08	0.32
Northeast	1	Ulster, NY	110.63	0.74	0.01	0.65	0.60	0.47
	2	King William, VA	23.89	0.84	0.04	0.27	0.77	0.67
	3	Putnam, NY	17.08	0.66	0.06	0.50	0.82	0.56
	4	Queen Anne’s, MD	12.44	0.53	0.07	0.52	0.66	0.74
	5	Albany, NY	10.00	0.72	0.12	0.57	0.95	0.32
	89	Baltimore city, MD	−0.81	0.34	0.44	0.21	0.09	0.17
	90	Bronx, NY	−1.60	0.63	0.43	0.16	0.11	0.19
	91	Salem, NJ	−1.65	0.81	0.12	0.15	0.15	0.69
	92	Somerset, MD	−1.75	0.63	0.08	0.14	0.01	0.86
	93	Arlington, VA	−1.90	0.46	0.21	0.11	0.46	0.01
	1	Kodiak Island, AK	44.20	0.41	0.01	0.42	0.54	0.71
North Pacific	2	Dillingham, AK	21.33	0.21	0.01	0.44	0.28	0.99
	3	Haines, AK	10.87	0.53	0.06	0.40	0.80	0.56
	4	Juneau City	6.62	0.57	0.16	0.33	0.99	0.82
	5	Aleutians West	4.12	0.55	0.12	0.44	0.42	0.74
	15	Yakutat City	0.13	0.27	0.56	0.12	0.62	0.57
	16	Hoonah-Angoon	0.10	0.17	0.23	0.31	0.29	0.59
	17	Wrangell City	−0.01	0.01	0.22	0.01	0.41	0.75
	18	Aleutians East	−5.91	0.42	0.02	0.24	0.19	0.51
	19	Bristol Bay	−7.54	0.91	0.09	0.01	0.23	0.59
Northeast	1	Hancock, ME	139.09	0.92	0.01	0.57	0.64	0.82
	2	Waldo, ME	57.07	0.90	0.01	0.30	0.59	0.67
	3	Dukes, MA	36.04	0.26	0.01	0.46	0.88	0.77
	4	Knox, ME	24.96	0.53	0.03	0.29	0.93	0.81
	5	Lincoln, ME	5.13	0.99	0.21	0.19	0.99	0.67
	24	New London, CT	0.11	0.08	0.11	0.65	0.01	0.52
	25	Strafford, NH	0.02	0.35	0.74	0.41	0.33	0.41
	26	Bristol, RI	0.00	0.01	0.51	0.01	0.63	0.72
	27	Suffolk, MA	−0.17	0.32	0.52	0.34	0.18	0.47
	28	Nantucket, MA	−0.22	0.36	0.18	0.41	0.14	0.53
Pacific	1	Honolulu, HI	28.69	0.99	0.39	0.91	0.99	0.99
	2	Hawaii, HI	3.99	0.70	0.99	0.99	0.55	0.48
	3	Maui, HI	2.37	0.92	0.27	0.50	0.38	0.10
	4	Kauai, HI	2.02	0.69	0.17	0.01	0.97	0.08
	5	Kalawao, HI	−1.79	0.01	0.01	0.05	0.01	0.01
Southeast	1	Pender, NC	17.20	0.58	0.05	0.56	0.61	0.48
	2	Camden, GA	10.73	0.99	0.12	0.32	0.65	0.69
	3	Bryan, GA	8.35	0.49	0.09	0.45	0.72	0.58
	4	Currituck, NC	7.54	0.62	0.13	0.45	0.48	0.82
	5	Carteret, NC	7.50	0.65	0.16	0.42	0.53	0.99
	49	Hertford, NC	−1.55	0.80	0.12	0.17	0.37	0.35
	50	Putnam, FL	−2.09	0.24	0.06	0.43	0.02	0.22
	51	Charlton, GA	−6.92	0.66	0.08	0.01	0.15	0.43
	52	Brantley, GA	−17.88	0.51	0.04	0.14	0.01	0.20
	53	Wayne, GA	−30.91	0.47	0.01	0.08	0.43	0.19
West	1	Clallam, WA	71.13	0.63	0.01	0.38	0.82	0.62
	2	Santa Cruz, CA	17.31	0.49	0.04	0.32	0.65	0.99
	3	Skagit, WA	14.15	0.84	0.10	0.43	0.72	0.97
	4	Whatcom, WA	13.34	0.70	0.10	0.54	0.80	0.90
	5	Marin, CA	12.73	0.39	0.04	0.31	0.75	0.92
	43	San Joaquin, CA	0.00	0.67	0.43	0.58	0.49	0.03
	44	Del Norte, CA	−0.04	0.71	0.32	0.08	0.26	0.77
	45	Columbia, OR	−1.25	0.99	0.27	0.22	0.41	0.32
	46	Pacific, WA	−2.70	0.72	0.12	0.12	0.40	0.39
	47	Wahkiakum, WA	−4.72	0.94	0.23	0.01	0.01	0.43

The top five (highest) and bottom five (lowest) scores are shown.

**TABLE 2 |:** Location Quotients for the Gulf of Mexico for Total Ocean Economy (OE)>1.0 and the ocean sectors location quotients for the ocean sectors: Living Resources (LR), Marine Construction (MC), Marine Transportation (MT), Offshore Mining and Extraction (OME), Ship and Boat Building (SBB) and Tourism and Recreation (TR).

		LQ							
		
County/Parish	State	OE	LR	MC	MT	OME	SBB	TR	CRSI
Monroe*	FL	4.29	1.02	0.24	0.02		0.05	1.72	0.35
Jackson**	MS	4.08		0.39			1.13	0.42	7.54
Refugio	TX	3.27				3.31		0.64	4.99
Terrebonne*	LA	3.09	2.79	1.49	0.46	2.03	5.79	0.53	1.38
Lafourche*	LA	3.00			6.50	0.63		0.10	1.63
Aransas	TX	2.99				1.05		1.32	5.48
San Patricio**	TX	2.83				0.17		0.69	13.24
Walton**	FL	2.69	0.29					1.72	5.91
Vermilion	LA	2.50	14.99					0.49	3.06
Plaquemines*	LA	2.47	0.83	2.39	2.70	2.74	2.32	0.24	0.49
St. Mary	LA	2.35		1.80	0.59		12.28	0.18	2.99
Iberia*	LA	2.35	1.92		0.62	2.73	3.63	0.47	0.47
Bay**	FL	2.19		0.28	0.03			1.50	13.50
Baldwin**	AL	2.12		0.42	0.39			1.63	9.83
Orleans*	LA	1.87	0.44		1.04	0.39	0.02	1.40	0.35
Galveston	TX	1.78	2.37	0.64	1.03	0.19		1.35	3.31
Franklin**	FL	1.75						1.75	64.49
Santa Rosa	FL	1.74		0.44		0.01		1.66	3.43
Okaloosa	FL	1.72	0.05	0.04				1.71	4.13
Harrison	MS	1.67	3.86	0.19	0.43			1.46	4.26
Matagorda**	TX	1.65	0.62					1.12	9.83
Jackson**	TX	1.62				0.37		0.62	11.56
Collier	FL	1.54	0.40	0.26	0.02			1.73	4.07
Charlotte	FL	1.48	0.64	0.68	0.03	0.04		1.69	3.29
Nueces**	TX	1.46			0.29	1.05		1.23	8.24
Jefferson	LA	1.43	0.75	0.55	1.04	0.18		1.35	2.67
Manatee*	FL	1.38	0.27	0.34	0.19			1.50	1.67
Willacy	TX	1.31						1.34	4.35
Escambia	FL	1.28	2.06	0.13	0.22			1.63	2.93
Lee	FL	1.27	0.63	0.64	0.13	0.02	0.17	1.68	2.24
Calhoun	TX	1.27		20.76	0.28			0.93	4.43
Wakulla	FL	1.21						1.75	5.22
Pinellas	FL	1.20	0.93	0.39	0.78		0.89	1.57	3.18
Kleberg*	TX	1.19						1.75	−0.35
Mobile	AL	1.19	5.50	0.99	1.38	0.01	17.12	0.71	3.27
Sarasota	FL	1.18	0.07	0.58	0.23			1.66	2.76
Chambers	TX	1.14				1.07		0.65	2.05
Levy**	FL	1.12	3.08					1.12	9.33
Gulf**	FL	1.10						1.75	25.60
Hernando	FL	1.06						1.30	4.02
Hancock*	MS	1.05			0.37			1.54	1.22
St. John the Baptist*	LA	1.04	1.41		1.56			0.00	1.72

* ranked in the bottom 10 CRSI scores

** ranked in the top 10 CRSI scores for the Gulf of Mexico Region ocean economy dependent counties.

## Footnotes

1
https://coast.noaa.gov/digitalcoast/training/enow-counties-list.html

2
https://www.fema.gov/coastal-high-hazard-area

3 Definitions from NOAA’s ENOW Explorer Tool: https://coast.noaa.gov/enowexplorer

4 Cross-walk of ocean sectors and NAICS industry codes: https://coast.noaa.gov/data/digitalcoast/pdf/enow-crosswalk-table.pdf

5 NOAA’s ENOWAPI: https://coast.noaa.gov/api/enow/

6 Count- level total employment data were obtained from the Bureau of Labor Statistics API: https://api.bls.gov/publicAPI/v2/timeseries/data/
